# Caffeine Administration Mitigates Chronic Stress-Induced Behavioral Deficits, Neurochemical Alterations, and Glial Disruptions in Rats

**DOI:** 10.3390/brainsci13121663

**Published:** 2023-11-30

**Authors:** Oritoke M. Okeowo, Olanrewaju O. Oke, Gloria O. David, Omamuyovwi M. Ijomone

**Affiliations:** 1 Department of Physiology, School of Basic Medical Sciences, Federal University of Technology, Akure 340252, Ondo State, Nigeria; omokeowo@futa.edu.ng (O.M.O.); lanreoke01@gmail.com (O.O.O.); 2 Laboratory for Experimental and Translational Neurobiology, University of Medical Sciences, Ondo 351101, Ondo State, Nigeria; 3 Department of Anatomy, School of Basic Medical Sciences, Federal University of Technology, Akure 340252, Ondo State, Nigeria; davidgloria4jesus@gmail.com; 4 Department of Anatomy, Faculty of Basic Medical Sciences, University of Medical Sciences, Ondo 351101, Ondo State, Nigeria

**Keywords:** caffeine, unpredictable chronic mild stress, behavior, GFAP, Iba-1

## Abstract

Prolonged exposure to stress has detrimental effects on health, and the consumption of caffeine, mostly contained in energy drinks, has become a widely adopted stress coping strategy. Currently, there is limited information regarding the effects of caffeine intake on chronic stress exposure. Thus, this study investigated the effects of caffeine administration on chronic stress-induced behavioral deficits, neurochemical alterations, and glial disruptions in experimental rats. Thirty male Wistar rats were randomly assigned to five groups (n = 6): non-stress control, stress control, and caffeine groups of doses 12.5, 25, and 50 mg/kg. The stress control and caffeine groups were subjected to an unpredictable chronic mild stress (UCMS) protocol daily for 14 days. The rats were evaluated for phenotypic and neurobehavioral assessments. Thereafter, the rat brains were processed for biochemical and immunohistochemical assays. Caffeine administration was found to ameliorate behavioral dysfunctions in rats exposed to UCMS. The UCMS-induced changes in brain levels of monoamines, cholinesterases, and some oxidative stress biomarkers were reversed by caffeine. Caffeine administration also produced mild protective effects against UCMS-induced changes in GFAP and Iba-1 expression in stress-specific brain regions. These results showed that low and moderate doses of caffeine reversed most of the stress-induced changes, suggesting its ameliorative potential against chronic stress-induced alterations.

## 1. Introduction

Stress refers to a state of threatened homeostasis. It occurs following exposure to extrinsic or intrinsic adverse forces (stressors) [1]. In today’s fast-paced and rapidly changing world, more people are exposed to stress, which is reported to affect one-third of the global population [2]. The source of stress could stem from work demands, financial strain, and the pressure to succeed in a highly competitive environment. Prolonged exposure to stress has serious health complications, ranging from mood disorders to cardiovascular diseases and behavioral dysfunctions [3].

Chronic stress is characterized by repeated exposure to different stressors and a maladaptation of coping mechanisms [4]. The activity of the HPA axis becomes dysregulated and glucocorticoids are produced in excessive amounts, resulting in immunosuppression and damage to multiple organs in the long term [3]. It corresponds with the exhaustion phase of General Adaptation Syndrome, where there is an increased risk of pathological state and failure of body systems [5]. Symptoms of this stage include burnout, fatigue, weakened immune system, reduced stress tolerance, anxiety, and depression [6].

Various brain regions such as the hippocampus, prefrontal cortex, and amygdala have been linked to stress-induced depressive states and changes in neuronal structure and functions [7]. Likewise, oxidative stress, characterized by increased reactive oxygen species, impacts neuronal integrity [8], while astroglial cells undergo morphological and functional changes, potentially influencing neurotransmitter balance and synaptic plasticity [9] as microglia activities become less efficient, resulting in superoxide and cytokine release [10]. The liver and kidneys are both susceptible to oxidative injury resulting in functional alterations, inflammatory response, and increased oxidative stress. Extended exposure to stress and glucocorticoids can also modify renal function, resulting in elevated blood pressure and constricted renal blood vessels. Stress hormones can also impact the liver, an important organ in metabolic regulation, which may impact glucose metabolism and perhaps result in insulin resistance [11]. To avert chronic stress, a significant proportion of the population has turned towards the intake of caffeine-containing products to provide short-term relief against feelings of stress. Caffeine being a psychostimulant is known to improve alertness, sustain intellectual efforts, and cause arousal during low mood states [12]. While the aforementioned strategy has become widely adopted, there has been little evidence from research to validate its effects against chronic stress-induced changes. Hence, this study aims to investigate the effects of caffeine intake on chronic stress-induced alterations with a focus on behavior, cognition, organ functions, and oxidative stress markers.

## 2. Materials and Methods

### 2.1. Experimental Animals

A total of 30 male Wistar rats (6 weeks old) of weights ranging from 80 to 120 g were used in this study. They were housed in clean plastic cages with sawdust bedding at room temperature. Food and water were available ad libitum except during periods of stress exposure. The rats were subjected to an acclimatization period of 7 days before the start of the experiment.

### 2.2. Drug Treatment

Lab-grade caffeine (Loba Chemie PVT Ltd., Mumbai, India) in the form of white anhydrous powder was used for this study. Desired doses of caffeine were prepared by dissolving calculated amounts of the drug in a specific volume of distilled water. The stress groups, except for the control, received their respective doses of caffeine (12.5, 25, and 50 mg/kg) daily throughout UCMS exposure. The drug was administered orally with the aid of an oral gavage and was carried out 30 min before stress exposure.

### 2.3. Unpredictable Chronic Mild Stress (UCMS) Protocol

The UCMS protocol was performed using the methods we have previously described [13] with minor modifications. It involves exposure of the rats to a variety of mild stressors. The stressors were randomly scheduled over the 2-week experiment, as shown in Table 1. The rats were subjected to 2 stressors per day except for the foreign object stressor which occurred for 24 h. On days where two stressors were carried out, one was scheduled for the morning session and the other for the afternoon session. The control group was not subjected to this protocol; they were left undisturbed in their cages except during routine procedures such as cage cleaning.

**Figure 1:**
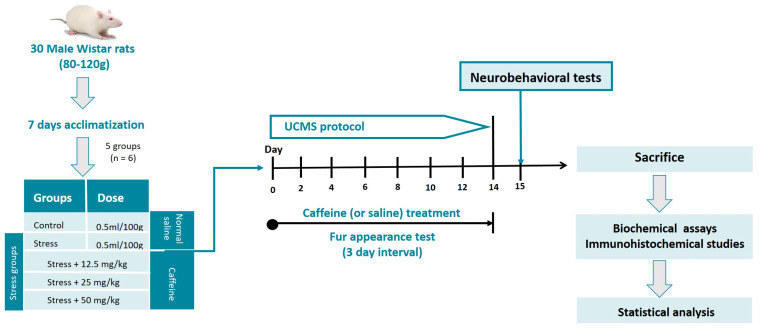
Experimental workflow.

All behavioral tests commenced 24 h after the last stressor of the UCMS protocol and were carried out between 8 am and 12 noon daily (see Figure 1). All apparatus was cleaned with 70% ethanol, dried with a paper towel, and then we waited for complete ethanol evaporation before placing the next rat for testing. This is essential to disinfect the apparatus and eliminate olfactory cues present in the apparatus.

### 2.4. Neurobehavioral Tests for Depression

#### 2.4.1. Fur Appearance Test

The fur appearance of the rats was assessed every three days throughout the UCMS period, as described by Aluko and Umukoro [13], with some modifications. Several body parts, including the head, neck, abdomen, tail, forepaws, hind paws, and genital regions were examined to assess the physical condition of the rat. Based on the conditions of the coat state, a score of 0, 0.5, or 1 was given for normal, medium, and poor states, respectively. The scores for the different body parts were summed up to obtain a total score and the average score was derived for representation.

#### 2.4.2. Sucrose Preference Test

The sucrose preference test was performed using the method of Liu et al. [14] with some minor modifications. The rats were deprived of food and water 24 h before the experiment. On the scheduled day of the experiment, the rats were housed separately in individual cages with access to 2 bottles containing 100 mL solution (1% *w*/*v*) and 100 mL of water. After one hour, the volume of consumed sucrose solution and water was measured and recorded, and sucrose preference was calculated using the appropriate formula.

#### 2.4.3. Forced Swim Test

The method used for the forced swim test was adapted from that of Aluko and Umukoro [13]. The rats were forced to swim for five minutes in a transparent plastic cylinder (50 cm in height and 25 cm in diameter) containing fresh water at room temperature to a depth of 30 cm, such that the rats could not touch the bottom. An overhead camera recorded the behaviors of the rats for five minutes; they were assessed for active swimming time, immobility time, and mobility time, which have been defined previously by Aluko and Umukoro [13]. Active swimming time is the time of swimming before the first pause of swimming activity, mobility time is the total time spent in swimming during the test phase, and immobility time is the total time spent immobile during the test phase.

### 2.5. Neurobehavioral Tests for Anxiety

#### 2.5.1. Elevated plus Maze (EPM) Test

This test was performed according to the method of Aluko and Umukoro [15] with some modifications. The rat was positioned in the central square of the maze facing the open arm and was allowed to explore the apparatus for 5 min. The behaviors of the rats were recorded with an overhead camera and the rats were assessed for open-arm duration, closed-arm duration, and latency time, which have been defined in a previous study [15].

#### 2.5.2. Light and Dark Test (LDT)

The test was performed as described by Arrant et al. [16] with some modifications. After a habituation period of two minutes, the rat was placed in the white compartment of the apparatus and allowed to explore the apparatus while the overhead camera recorded its movements for five minutes. The parameters recorded included the duration in the dark compartment, the duration in the light compartment, and the latency of the first crossing. Entry into the other compartment was considered when all four paws were placed in the opposite compartment.

### 2.6. Neurobehavioral Tests for Memory

#### 2.6.1. Y-Maze Test

The Y-maze test assesses spontaneous alternation performance as a measure of working memory in rats. The Y-maze was designed as described by Eduviere et al. [17] with minor modifications. The apparatus was constructed out of wooden material as a Y-shaped arena with three arms of identical length orientated at 120 angles from each other. The rats were tested in the light phase of their cycle. After an acclimatization period of two minutes, the rat was placed in the start arm of the maze with the arms labeled A, B, and C, and their behavior was recorded with an overhead camera for five minutes. Then, the number of arm entries and alternations was used to calculate the percentage alternation by the appropriate formula. An alternation during the Y-maze test is defined as correct when the rat chooses to enter a different arm from the previous entry.

#### 2.6.2. Object Recognition Test (ORT)

The object recognition test assesses memory functions in rats and relies on the rat’s natural proclivity for exploring novel objects. The test was performed according to the method of Aluko and Umukoro [15]. The test took place in a sound-isolated room illuminated by natural light. The objects to be discriminated against were made of wooden material and their weight was such that they could not be displaced by the rats. The time spent exploring the novel object and familiar object were recorded and the preference score was calculated for each rat. The preference score is the fraction of time spent with a novel object against the familiar object. It was expressed as a percentage.

### 2.7. Neurobehavioral Tests for Locomotion

#### 2.7.1. Wire Grip Test

The wire grip test evaluates motor function and deficit. It is also used to detect neuromuscular abnormalities of muscle strength in rats. The procedure used was based on that of Van Putten et al. [18] with some modifications. A multi-stranded twisted wire was secured between two vertical stands, with the wire positioned at 60 cm above ground level to ensure that the rats could not touch the ground with their tails. Wood shavings were placed below the wire to cushion their fall. The rat was made to grasp the middle of the wire by the forelimbs only. As soon as the rat was suspended, a timer was started, the total hanging time was recorded, and each rat was graded based on their ability to complete the wire grip test as previously classified by Van Putten et al. [13].

#### 2.7.2. Beam Walk Test

The beam walk test assesses balance and fine motor coordination. The test was performed based on that of Drucker-Colin [19]. A wooden beam of 2.5 cm in width with a length of 1 meter was suspended 70 cm above the ground by 2 wooden supports, and a goal box with a small opening was located at one end of the beam. The apparatus was situated in a sound-isolated room illuminated by natural light. For each trial, the rat was placed on the surface of the start-end of the beam opposite the goal box and allowed to cross the beam to enter the goal box at the other end. The number of foot slips was counted, the time to enter the box was recorded with a stopwatch, and each rat’s performance on the beam walking test was graded as previously described by Drucker-Colin [14].

### 2.8. Sacrifice, Sample Collection, and Preparation

The rats were sacrificed 24 h after the neurobehavioral tests by cervical dislocation. The brain, kidney, and liver were harvested, rinsed with normal saline, weighed, and stored in sample bottles containing the appropriate physiological solutions. The brain samples were further processed for biochemical and immunohistochemistry quantifications. Blood samples were obtained from the rats’ tails and via the retro-orbital route to determine blood glucose and serum corticosterone levels, respectively.

### 2.9. Determination of Blood Glucose and Serum Corticosterone Levels

The blood glucose level was measured by using a glucometer. Blood samples were obtained from the tail of the rats and the glucose level was read using a commercially available glucometer strip (Accu-Chek Actives, Roche Diagnostics India Pvt., Ltd., Maharashtra, India). Corticosterone determination was performed in serum with a commercial ELISA kit (Oxford Biomedical Research, Rochester, Michigan, USA) according to the manufacturer’s instructions.

### 2.10. Determination of Neurotransmitter Activity

Monoamine (NE, DA, and 5-HT) concentrations were evaluated in brain samples by using ELISA kits (Ad Literan Diagnostic Labs, Indianapolis, IN, USA) according to the manufacturer’s instructions. Acetylcholinesterase and butyrylcholinesterase activities were determined according to the spectrophotometric method described by Ellman et al. [20] and their activities were expressed as nmol/mg protein.

### 2.11. Assessment of Kidney and Liver Functions

Various serum assays for the kidney and liver function tests were performed. For the kidneys, determination of creatinine was conducted using the technique described by Heinegård and Tiderström [21]. A uric Acid Assay kit (BioAssay Systems, San Francisco, CA, USA) was used to measure uric acid levels according to standard procedure [22]. Determination of urea levels was conducted utilizing the Urease–Berthlot method. For the liver, Alanine aminotransferase (ALT) and aspartate aminotransferase (AST) levels were quantified according to the method outlined by Reitman and Frankel [23]. Determination of the alkaline phosphatase (ALP) level was based on the method of Wright et al. [24].

### 2.12. Protein Determination

The protein level in the brain samples was quantified using the Bradford method, which is based on the principle of protein–dye binding [25].

### 2.13. Determination of Biomarkers of Oxidative Stress

Catalase activity was quantified in the supernatant fraction of the brain homogenate in a method previously described by Sinha [26]. Superoxide dismutase (SOD) activity was determined using the technique outlined by Misra and Fridovich [27]. The activity of Glutathione S-transferase (GST) was determined using the method of Moron et al. [28]. Glutathione peroxidase (GPx) activity was estimated using the method described by Maral et al. [29]. The activities of catalase, SOD, GPx, and GST were expressed as µmol·L^−1^. The nitrite content was determined using the Greiss reaction [30]. The nitrate levels were measured according to Bories and Bories [31]. The brain concentration of MDA, a biomarker of lipid peroxidation, was determined according to the method of Ohkawa et al. [32]. The level of reduced GSH in the brain supernatant was determined by the method described by Moron, Depierre, and Mannervik [28]. The determination of the total antioxidant capacity was performed utilizing the BioVision™ Total Antioxidant Capacity Assay (TAC) (Bioptics, Tucson, AZ, USA). An assay for H^+^/K^+^ ATPase activity was performed as described by Wallmark et al. [33]. Na^+^/K^+^-ATPase and Ca^2+^-ATPase activities were assayed by the method of Bonting [34] and Hjertén and Pan [35], respectively.

### 2.14. GFAP and Iba-1 Immunohistochemistry

Immunohistochemical studies for glial fibrillary acidic protein (GFAP) and ionized calcium-binding adapter molecule 1 (Iba-1) were performed to assess astroglial and microglial activity in stress-responsive brain regions using our established protocols [36]. The following brain regions were accessed with the rat brain atlas as a reference [37]: the prefrontal cortex (Bregma 2.76 to −3.24 mm), the hippocampus (Bregma −3.00 to −3.60 mm), the thalamus (Bregma −3.00 to −3.60 mm), and the cerebellum (Bregma −12.48 to −12.84 mm). In brief, sections of 5 µm thickness obtained from routine paraffin were deparaffinized and subjected to antigen retrieval by heating in a citrate-based antigen unmasking solution of pH 6.0 (Vector Labs, Newark, CA, USA) for 30 min in a steamer. Endogenous peroxidase blocking was performed in 0.3% hydrogen peroxide in phosphate-buffered saline (PBS, pH 7.4) for 10 min. Sections were then incubated at room temperature for 2 h in primary rabbit antibodies GFAP (ThermoFisher, Waltham, MA, USA; #16825-1-AP) at 1:7500, and IBA1 (Cell Signalling, Danvers, MA, USA; #17198) at 1:1250. Sections were washed in PBS and incubated in ImmPRESS™ HRP Anti-Rabbit IgG (Peroxidase) Polymer Reagent, made in horses (Vector Labs, USA). Color was developed with a DAB Peroxidase (HRP) Substrate Kit (Vector Labs, USA), and sections were counter-stained in hematoxylin. Immunostained slides were digitized with the Pannoramic 250 Flash II slide scanner (3D Histech, Budapest, Hungary). A total of 5 to 10 random non-overlapping photomicrographic fields of the aforementioned brain regions were captured at ×400 magnification using the accompanying digital microscopy platform, CaseViewer. Digital images were imported into Image J software (1.47 version, Bethesda, MD, USA) and analyzed using the cell counter tool and ImmunoRatio plugins.

### 2.15. Data Management and Statistical Analysis

Statistical analysis was performed using Graph Pad Prism Software version 9.3.0 (Boston, MA, USA). The data obtained were presented as group mean ± SEM. Data derived from the fur appearance test were subjected to a two-way ANOVA test while the data from other tests were subjected to a one-way ANOVA test, which was followed up with the Tukey post hoc test. A *p*-value of less than 0.05 was chosen as the significance level for all statistical analyses.

## 3. Results

### 3.1. Caffeine Administration Ameliorated Depressive-like Behaviors in Rats Exposed to UCMS

**Figure 2:**
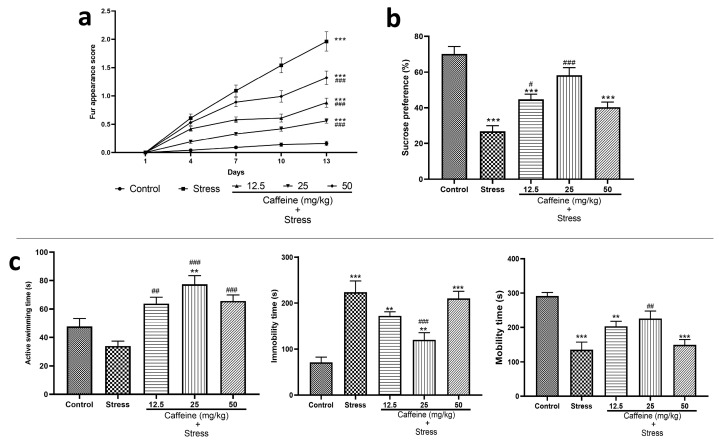
Comparison of the influence of caffeine administration on UCMS-induced depression in rats. (**a**) Fur appearance. (**b**) Sucrose preference. (**c**) Forced swim test. The stress group had increased fur appearance score, immobility time, decreased active swimming time, and sucrose preference, which were reversed by caffeine administration. Each bar denotes group mean ± SEM. ** *p* < 0.01, *** *p* < 0.001 vs. control; # *p* < 0.05, ## *p* < 0.01, ### *p* < 0.001 vs. stress. ANOVA test with Tukey’s post hoc test.

In the fur appearance test, two-way ANOVA showed a significant difference between the groups for fur appearance score (F (4, 125) = 148.5; *p* < 0.0001). Post hoc analysis shows that UCMS significantly increased the fur appearance score when compared to the control. However, this effect was reversed by caffeine (12.5, 25, and 50 mg/kg) as it produced a significant decrease in the fur appearance score compared to the stress group (Figure 2a). The decreased fur appearance score indicates an improved coat state of the rats.

The result of the sucrose preference test is shown in Figure 2b. One-way ANOVA revealed a significant difference between groups (F (4, 25) = 22.54; *p* < 0.0001). Post hoc analysis shows that UCMS significantly decreased sucrose preference compared to the control. This effect was reversed by caffeine (12.5 and 25 mg/kg) as it significantly increased sucrose preference compared to the stress group.

Figure 2c shows the effects of caffeine administration on performance in the forced swim test. One-way ANOVA showed a significant difference between the treatment groups: active swimming time (F (4, 25) = 12.01; *p* < 0.0001), mobility time (F (4, 25) = 13.16; *p* < 0.0001), and immobility time (F (4, 25) = 16.01; *p* < 0.0001).

Post hoc analysis shows that UCMS significantly decreased the mobility time stress control when compared with the control (*p* < 0.0001). UCMS also caused a significant increase in the immobility time compared to the control group (*p* < 0.0001). Caffeine administration reversed these effects as the 25 mg/kg dose increased the mobility time and decreased the immobility time in comparison to the stress group. Caffeine (12.5, 25, and 50 mg/kg) increased active swimming time compared to the stress group.

### 3.2. Caffeine Administration Protected against Anxiety-like Behaviors in UCMS-Exposed Rats

**Figure 3:**
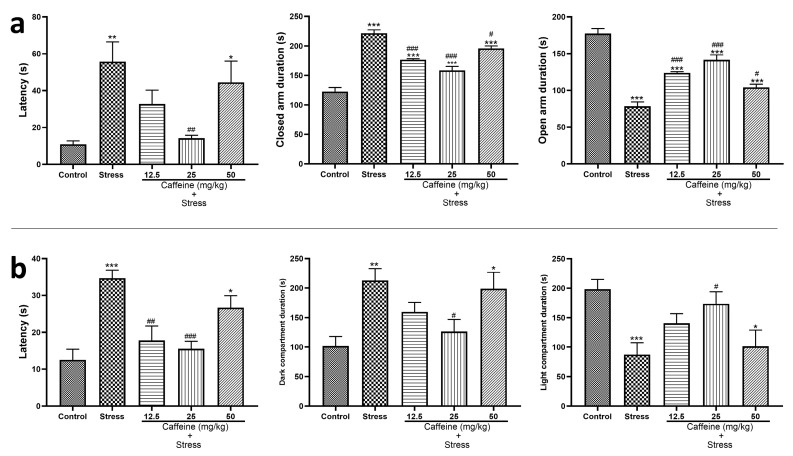
Comparison of the influence of caffeine administration on UCMS-induced anxiety in rats. The stress group had increased EPM and LDT latency, and increased duration in the dark compartment and closed arm, which were reversed by caffeine administration. (**a**) Elevated plus maze test. (**b**) Light and dark test. Each bar denotes group mean ± SEM. * *p* < 0.05, ** *p* < 0.01, *** *p* < 0.001 vs. control; # *p* < 0.05, ## *p* < 0.01, ### *p* < 0.001 vs. stress. One-way ANOVA test with Tukey’s post hoc test.

In the EPM result displayed in Figure 3a, one-way ANOVA showed a significant difference between the groups: transfer latency (F (4, 25) = 5.97; *p* < 0.0001), duration in the closed arm (F (4, 25) = 47.95; *p* < 0.0001), and duration in open arm (F (4, 25) = 47.95; *p* < 0.0001).

The UCMS-exposed group had increased transfer latency, increased duration in the closed arm, and decreased duration in the open arm when compared to the control. However, caffeine reversed these UCMS-induced changes. Caffeine (25 mg/kg) administration decreased transfer latency in comparison to the stress group while the caffeine (12.5, 25, and 50 mg/kg) increased the duration in the closed arm and decreased duration in the open arm in comparison to the stress group (Figure 3a).

The effect of caffeine administration on the light and dark test (LDT) performance is displayed in Figure 3b. One-way ANOVA showed a significant difference between the groups: latency (F (4, 25) = 9.57; *p* < 0.0001), duration in the dark compartment (F (4, 25) = 5.17; *p* = 0.0035), and duration in the light compartment (F (4, 25) = 5.17; *p* < 0.0035).

UCMS-exposed rats had an increased latency, longer duration in the dark compartment, and shorter duration in the light compartment compared with the control. These UCMS-induced changes were reversed by caffeine administration. Caffeine (25 mg/kg) decreased both latency and duration in the dark compartment. It also increased duration in the light compartment in comparison to the stress group (Figure 3b).

### 3.3. Caffeine Protected against UCMS-Induced Impairment of Memory Functions in Rats

**Figure 4:**
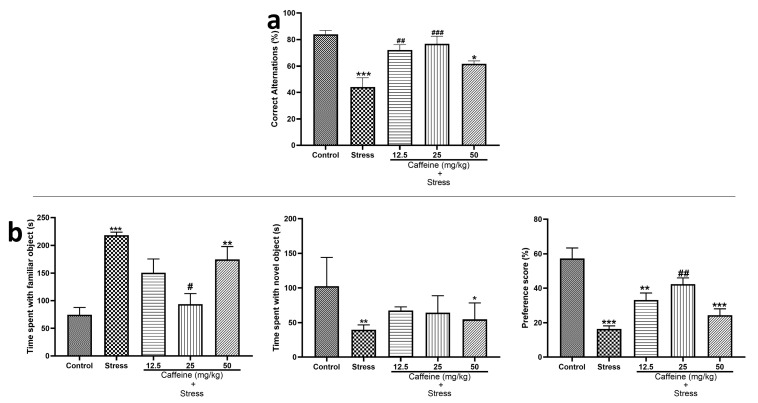
Comparison of the influence of caffeine administration on UCMS-induced changes in memory functions. There was a decreased percentage of correct alternations and preference scores in the stress group, which were reversed by caffeine administration. (**a**) Y-maze test. (**b**) Object recognition test. Each bar denotes group mean ± SEM. * *p* < 0.05, ** *p* < 0.01, *** *p* < 0.001 vs. control; # *p* < 0.05, ## *p* < 0.01, ### *p* < 0.001 vs. stress. One-way ANOVA with Tukey’s post hoc test.

Figure 4a shows the effects of caffeine administration on memory function in UCMS-exposed rats. One-way ANOVA revealed a significant difference between the groups for % correct alternation (F (4, 25) = 11.11; *p* < 0.0001) in the Y-maze test. UCMS significantly decreased the percentage of correct alternation in comparison to the control. Caffeine (12.5 and 25 mg/kg) improved memory performance in UCMS-exposed rats.

For ORT, which is displayed in Figure 4b, one-way ANOVA showed a significant difference between the treatment groups for time spent with a familiar object (F (4, 25) = 8.32; *p* = 0.0002), time spent with the novel object (F (4, 25) = 5.39; *p* = 0.0029), and preference score (F (4, 25) = 15.28; *p* < 0.0001). Post hoc analysis showed that the time spent with the familiar object was significantly increased in the stress group in comparison to the control. Also, the time spent with the novel object and the preference score in the stress were significantly decreased when compared to the control. A total dose of 25 mg/kg caffeine decreased the time spent with the familiar object; the same dose also produced an increased preference score when compared to the stress.

### 3.4. Caffeine Protects against UCMS-Induced Impairment of Locomotive Function

**Figure 5:**
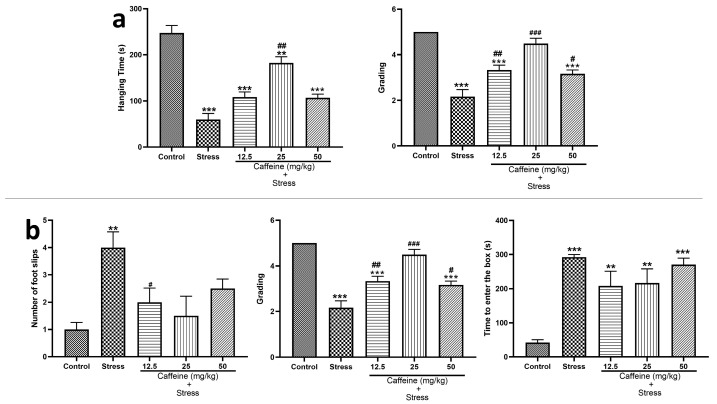
Comparison of the influence of caffeine administration on UCMS-induced changes in locomotion (**a**) Wire grip test. (**b**) Beam walking test. The stress groups had decreased hanging time and grading in the wire grip test, an increased number of foot slips, and time to enter to box in the beam walking test, which were reversed by caffeine administration. ** *p* < 0.01, *** *p* < 0.001 vs. control; # *p* < 0.05, ## *p* < 0.01, ### *p* < 0.001 vs. stress. One-way ANOVA with Tukey’s post hoc test.

In the wire grip test (Figure 5a), one-way ANOVA showed a significant difference between the groups for hanging time (F (4, 25) = 34.93; *p* < 0.0001) and grades (F (4, 25) = 29.29; *p* < 0.0001). UCMS caused a significant (*p* < 0.05) decrease in the hanging time and the grades when compared to the control. However, the 25 mg/kg caffeine dose significantly increased hanging time while caffeine (12.5, 25, and 50 mg/kg) significantly increased the grades compared to the stress group.

In the beam walking test shown in Figure 5b, one-way ANOVA showed a significant difference between the groups for the number of foot slips (F (4, 25) = 34.93; *p* < 0.0001), latency (F (4, 25) = 18.41; *p* < 0.0001), and time taken to enter to the box (F (4, 25) = 11.84; *p* < 0.0001). Post hoc tests showed that UCMS increased the number of foot slips, latency, and time taken to enter the box when compared to the control. Caffeine (12.5, 25, and 50 mg/kg) treatment decreased the number of foot slips and latency, respectively, in comparison to the stress group.

### 3.5. Caffeine Prevented UCMS-Induced Increase in Blood Glucose and Serum Corticosterone Levels in Rats

**Figure 6:**
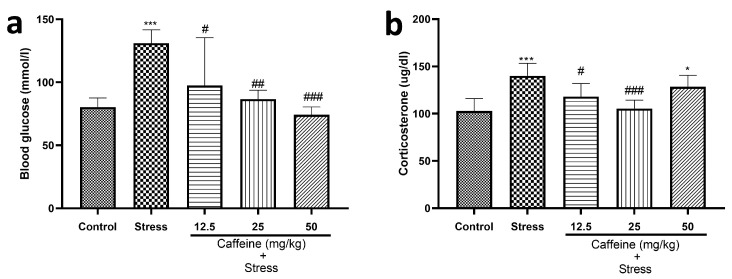
Comparison of the influence of caffeine administration on UCMS-induced changes in blood glucose and serum corticosterone levels. (**a**) Blood glucose level. (**b**) Corticosterone level. The stress group had elevated blood glucose and serum corticosterone levels, which were reversed by caffeine administration. Each bar denotes group mean ± SEM. * *p* < 0.05, *** *p* < 0.001 vs. control; # *p* < 0.05, ## *p* < 0.01, ### *p* < 0.001 vs. stress. One-way ANOVA with Tukey’s post hoc test.

One-way ANOVA indicated a significant difference between the groups for blood glucose level (F (4, 25) = 8.92; *p* = 0.0001) and corticosterone level (F (4, 25) = 9.67; *p* < 0.0001) (Figure 6).

UCMS elevated blood glucose and corticosterone levels in comparison to the control. Caffeine (12.5, 25, and 50 mg/kg) produced a significant decrease in blood glucose levels while 25 mg/kg of caffeine reduced corticosterone levels in comparison to the stress group.

### 3.6. Caffeine Reversed UCMS-Induced Changes in Brain Cholinesterase and Monoaminergic Levels in Rats

**Figure 7:**
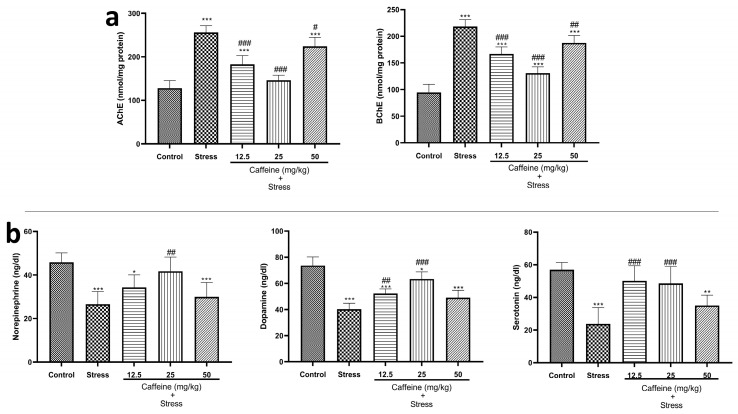
Comparison of the influence of caffeine administration on UCMS-induced changes in neurotransmitter activities. (**a**) Cholinesterase. (**b**) Norepinephrine, Dopamine, and Serotonin (5-HT). Elevated cholinesterase levels and reduced neurotransmitter levels in the stress group were reversed by caffeine administration. Each bar denotes group mean ± SEM. * *p* < 0.05, ** *p* < 0.01, *** *p* < 0.001 vs. control; # *p* < 0.05, ## *p* < 0.01, ### *p* < 0.001 vs. stress. One-way ANOVA with Tukey’s post hoc test.

One-way ANOVA showed a significant difference between the groups: acetylcholinesterase (F (4, 25) = 56.38; *p* < 0.0001) and butyrylcholinesterase (F (4, 25) = 78.60; *p* < 0.0001) (Figure 7a). Post hoc tests indicate that UCMS produced a significant increase in cholinesterase levels when compared to the control. Caffeine (12.5, 25, and 50 mg/kg) counteracted this effect. These doses produced a significant decrease in cholinesterase levels when compared to the stress group.

The effects of caffeine on monoaminergic levels in rats exposed to UCMS are shown in Figure 7b. One-way ANOVA revealed a significant difference between the treatment groups: NE (F (4, 25) = 11.18; *p* < 0.0001), DA (F (4, 25) = 36.56; *p* < 0.0001), and 5-HT (F (4, 25) = 15.25; *p* < 0.0001). Post hoc tests showed that UCMS caused a significant decrease in monoamine levels when compared to the control. Caffeine (25 mg/kg) produced a significant increase in NE levels while 12.5 and 25 mg/kg of caffeine significantly increased DA and 5-HT levels when compared to the stress group.

### 3.7. Caffeine Inhibited Alterations in Kidney Functions and Liver Enzyme Concentrations in UCMS-Exposed Rats

**Figure 8:**
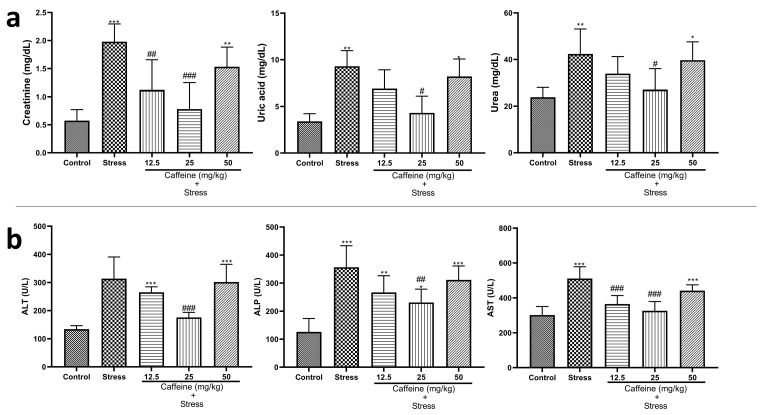
Comparison of the influence of caffeine administration on UCMS-induced changes in kidney and liver function tests. (**a**) Kidney functions. (**b**) Liver functions. The stress group had increased levels of creatinine, uric acid urea, and liver enzymes, which were reversed by caffeine administration. * *p* < 0.05, ** *p* < 0.01, *** *p* < 0.001 vs. control; # *p* < 0.05, ## *p* < 0.01, ### *p* < 0.001 vs. stress. One-way ANOVA with Tukey’s post hoc test.

One-way ANOVA showed a significant difference between the groups: creatinine (F (4, 25) = 12.55; *p* < 0.0001), uric acid (F (4, 25) = 9.67; *p* < 0.0001), and urea (F (4, 25) = 5.71; *p* = 0.0021) (Figure 8a). Post hoc analysis showed that UCMS increased creatinine, uric acid, and urea levels when compared to the control. However, all these changes were reversed by CA (25 mg/kg).

One-way ANOVA indicated a significant difference between the groups for liver enzyme concentration: ALT (F (4, 25) = 17.61; *p* < 0.0001), ALP (F (4, 25) = 14.09; *p* < 0.0001), and AST (F (4, 25) = 16.91; *p* < 0.0001) (Figure 8b). Post hoc analysis showed that UCMS significantly elevated ALP and AST levels in the stress group when compared to the control. Caffeine (25 mg/kg) reduced ALP and ALT levels while 12.5 and 25 mg/kg of caffeine reduced AST levels in rats exposed to UCMS.

### 3.8. Caffeine Administration Regulated Biomarkers of Oxidative Stress in UCMS-Exposed Rats

**Figure 9:**
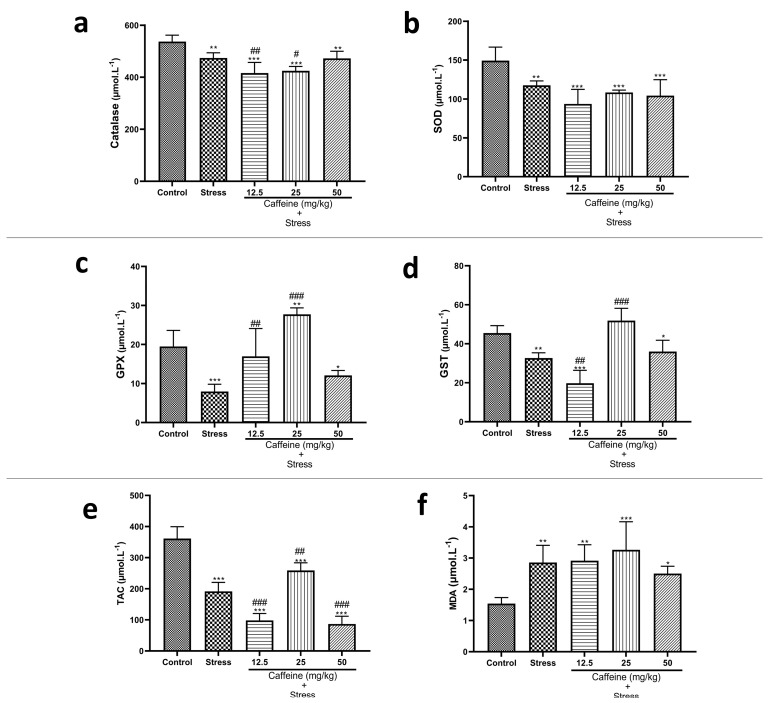
Comparison of the influence of caffeine administration on UCMS-induced changes in biomarkers of oxidative stress. (**a**) Catalase, (**b**) SOD, (**c**) GPx, (**d**) GST, (**e**) TAC, (**f**) MDA. The stress groups had decreased activities of antioxidant enzymes and total antioxidant capacity, which were reversed by caffeine administration, but there were no significant effects on MDA and SOD activities. Each bar denotes group mean ± SEM. * *p* < 0.05, ** *p* < 0.01, *** *p* < 0.001 vs. control; # *p* < 0.05, ## *p* < 0.01, ### *p* < 0.001 vs. stress. One-way ANOVA with Tukey’s post hoc test.

One-way ANOVA showed a significant difference between the groups: catalase (F (4, 25) = 18.99; *p* < 0.0001), TAC (F (4, 25) = 98.69; *p* < 0.0001), SOD (F (4, 25) = 12.10; *p* < 0.0001), GPx (F (4, 25) = 22.64; *p* < 0.0001), and GST (F (4, 25) = 32.66; *p* < 0.0001) (Figure 9). Post hoc analysis revealed that UCMS significantly decreased catalase, TAC, SOD, GPx, GST, Na^+^K^+^-ATPase, and Ca^2+^-ATPase activities when compared to the controls. UCMS also caused an elevation in MDA levels in comparison with the controls. Caffeine (25 mg/kg) reversed UCMS-induced changes in TAC, GPx, CAT, and GST activities. Caffeine administration produced no significant effect on SOD and MDA levels.

### 3.9. Caffeine Administration Modified the Levels of ATPases but Not Nitrite Concentrations in UCMS-Exposed Rats’ Brains

**Figure 10:**
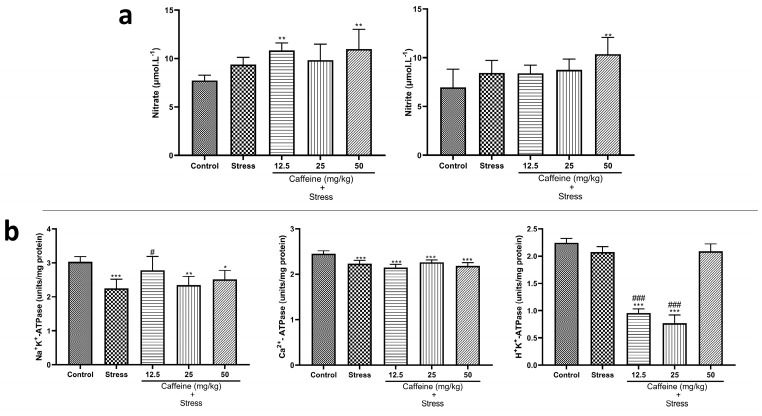
Comparison of the influence of caffeine administration on UCMS-induced changes in nitrites and ATPases (**a**) Nitrites (**b**) ATPases. Each bar denotes group mean ± SEM. * *p* < 0.05, ** *p* < 0.01, *** *p* < 0.001 vs. control; # *p* < 0.05, ### *p* < 0.001 vs. stress. One-way ANOVA with Tukey’s post hoc test.

One-way ANOVA showed a significant difference between the groups: nitrite (F (4, 25) = 17.61; *p* < 0.0001), nitrate (F (4, 25) = 4.44; *p* = 0.0075), Na^+^K^+^-ATPase (F (4, 25) = 7.67; *p* = 0.0004), Ca^2+^-ATPase (F (4, 25) = 18.09; *p* < 0.0001), and H^+^K^+^-ATPase (F (4, 25) = 228.4; *p* < 0.0001) (Figure 10). Post hoc analysis revealed that UCMS significantly decreased Na^+^K^+^-ATPase and Ca^2+^-ATPase levels when compared to the control. Caffeine (12.5 mg/kg) reversed UCMS-induced changes in Na^+^K^+^-ATPase levels. Caffeine treatment produced no significant effect on nitrite and nitrate levels.

### 3.10. Caffeine Protected against UCMS-Induced Astroglial Deficits in Rats’ Brains

**Figure 11:**
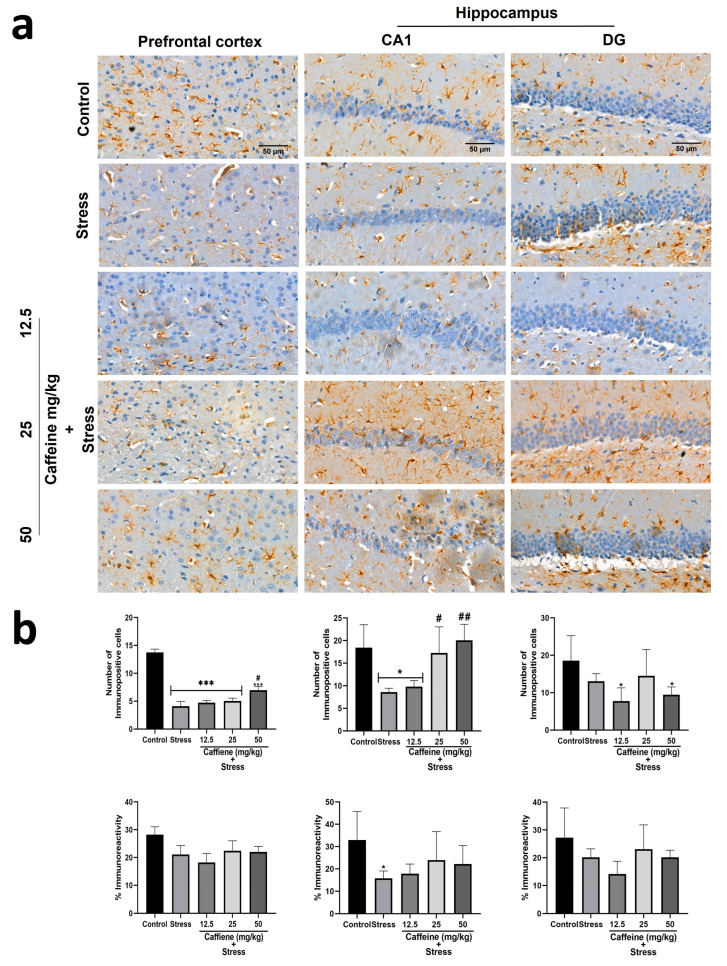
Comparison of the influence of caffeine administration on GFAP immunoreaction in the prefrontal cortex and hippocampus of UCMS-exposed rats. (**a**) Representative photomicrographs (magnification = ×400). (**b**) Bar graphs showing immunoreactivity (down) and the number of GFAP-positive cells (up). Each bar denotes group mean ± SEM. * *p* < 0.05, *** *p* < 0.001 vs. control; # *p* < 0.05, ## *p* < 0.01, vs. stress. One-way ANOVA with Tukey’s post hoc test.

In the prefrontal cortex, one-way ANOVA showed a significant difference between the groups for the number of immunopositive cells (F (4, 15) = 41.82; *p* < 0.0001) (Figure 11). Post hoc analysis showed that UCMS significantly decreased the number of GFAP-positive cells in comparison to the control. This effect was reversed by caffeine (50 mg/kg) administration. There was no significant difference in GFAP immunoreactivity (GFAP-IR) between the groups.

One-way ANOVA showed a significant difference between the groups: number of immunopositive cells in CA1 (F (4, 15) = 7.4; *p* = 0.0017), percentage of immunoreactivity in CA1 (F (4, 15) = 2.11; *p* = 0.13), and number of immunopositive cells in DG (F (4, 15) = 3.14; *p* = 0.0461) (Figure 11). In the CA1 zone, post hoc analysis showed that UCMS decreased the GFAP-positive cell count and GFAP-IR in comparison to the control. Caffeine (25 and 50 mg/kg) reversed UCMS-induced changes in the GFAP-positive cell count but not in GFAP-IR. In the DG, exposure to UCMS produced no significant effect on the GFAP-positive cell count and GFAP-IR.

**Figure 12:**
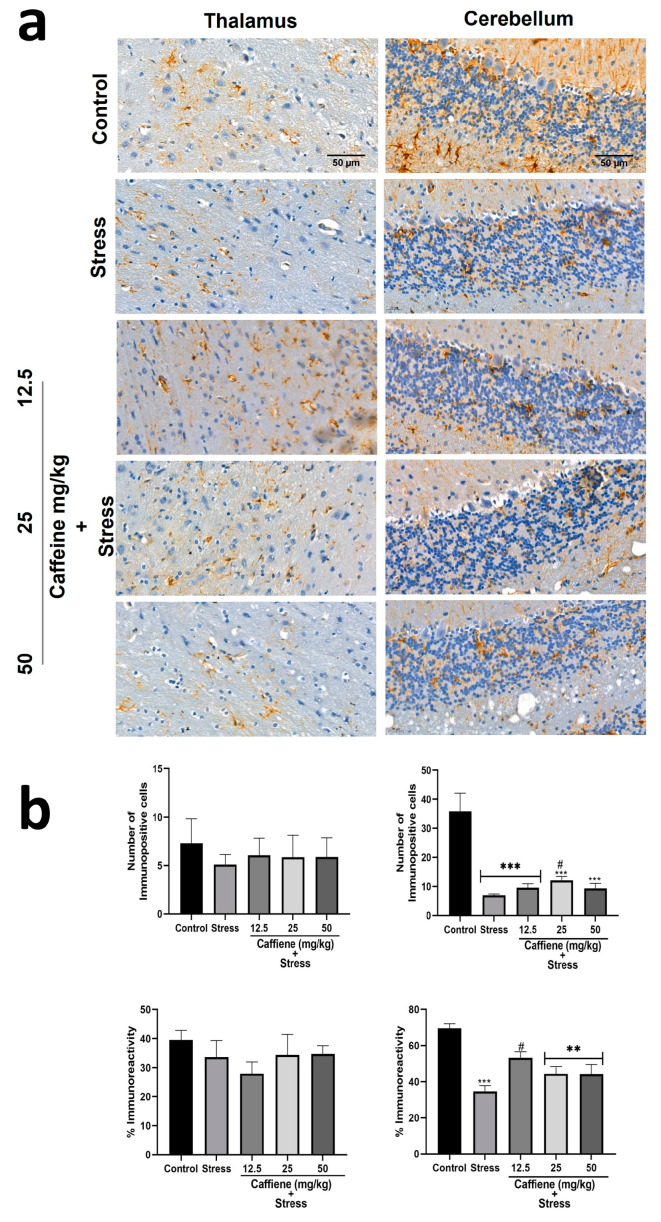
Comparison of the influence of caffeine administration on GFAP immunoreaction in the thalamus and cerebellum of UCMS-exposed rats. (**a**) Representative photomicrographs (magnification = ×400). (**b**) Bar graphs showing immunoreactivity (down) and the number of GFAP-positive cells (up). Each bar denotes group mean ± SEM. ** *p* < 0.01, *** *p* < 0.001 vs. control; # *p* < 0.05, vs. stress. One-way ANOVA with Tukey’s post hoc test.

In the cerebellum, one-way ANOVA showed a significant difference between the groups: number of immunopositive cells (F (4, 15) = 15.25; *p* < 0.0001) and percentage immunoreactivity (F (4, 15) = 12.04; *p* = 0.0001) (Figure 12). According to post hoc analysis, UCMS exposure decreased the GFAP-positive cell count and percentage immunoreactivity in comparison to the control. Treatment with 25 mg/kg caffeine and 12.5 mg/kg caffeine increased both the GFAP-positive cell count and percentage immunoreactivity, respectively, following exposure to UCMS. However, one-way ANOVA showed no significant difference between the groups in the thalamus (Figure 12).

### 3.11. Caffeine Protects against UCMS-Induced Microglial Deficits in Rats’ Brains

**Figure 13:**
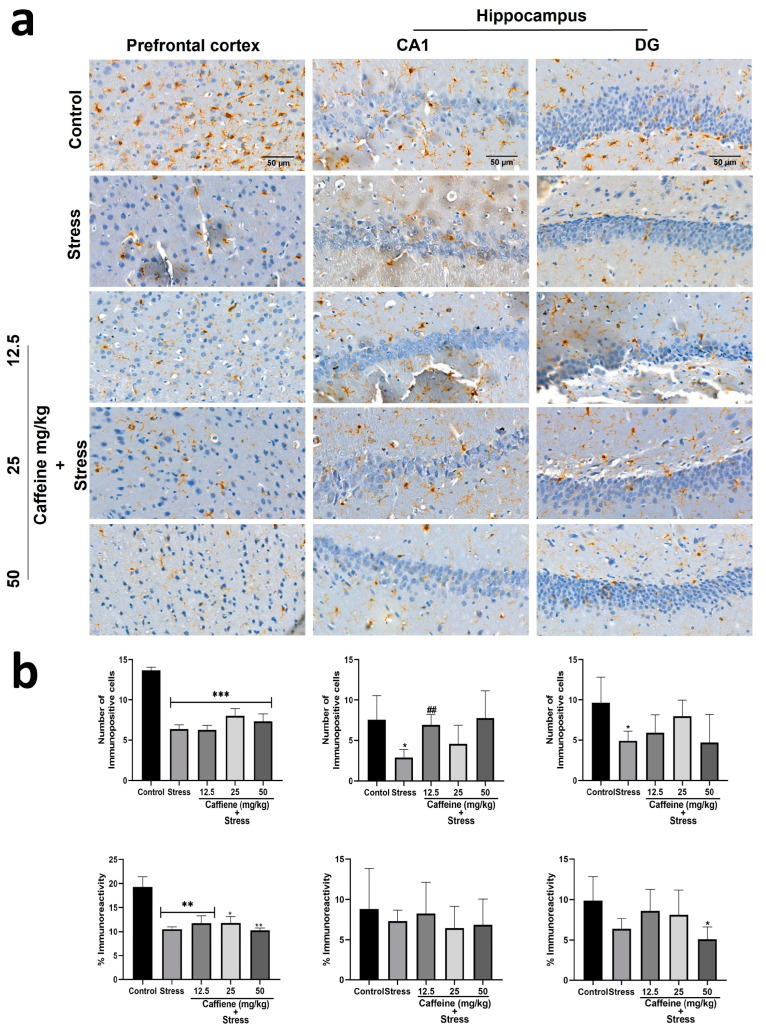
Comparison of the influence of caffeine administration on Iba-1 immunoreaction in the prefrontal cortex and hippocampus of UCMS-exposed rats. (**a**) Representative photomicrographs (magnification = ×400). (**b**) Bar graphs showing immunoreactivity (down) and the number of Iba-1-positive cells (up). Each bar denotes group mean ± SEM. * *p* < 0.05, ** *p* < 0.01, *** *p* < 0.001 vs. control; ## *p* < 0.01, vs. stress. One-way ANOVA with Tukey’s post hoc test.

In the prefrontal cortex, one-way ANOVA indicated a significant difference between the groups: number of immunopositive cells (F (4, 15) = 19.10; *p* < 0.0001) and percentage immunoreactivity (F (4, 15) = 7.64; *p* = 0.0015) (Figure 13). Post hoc analysis showed that UCMS decreased the number of Iba-1-positive cells and the percentage of immunoreactivity in comparison with the control. Caffeine treatment produced no significant effect on both the Iba-1-positive cell count and percentage of immunoreactivity.

In the hippocampus, one-way ANOVA showed a significant difference between the groups: the number of immunopositive cells in the CA1 (F (4, 15) = 3.204; *p* = 0.0434) and the number of immunopositive cells in the DG (F (4, 15) = 42.28; *p* < 0.0001) (Figure 13). Post hoc analysis showed that UCMS decreased the number of Iba-1 positive cells in the CA1 and DG in comparison to the control. Treatment with caffeine (12.5 mg/kg) increased the number of Iba-1-positive cells in the CA1 following exposure to UCMS.

**Figure 14:**
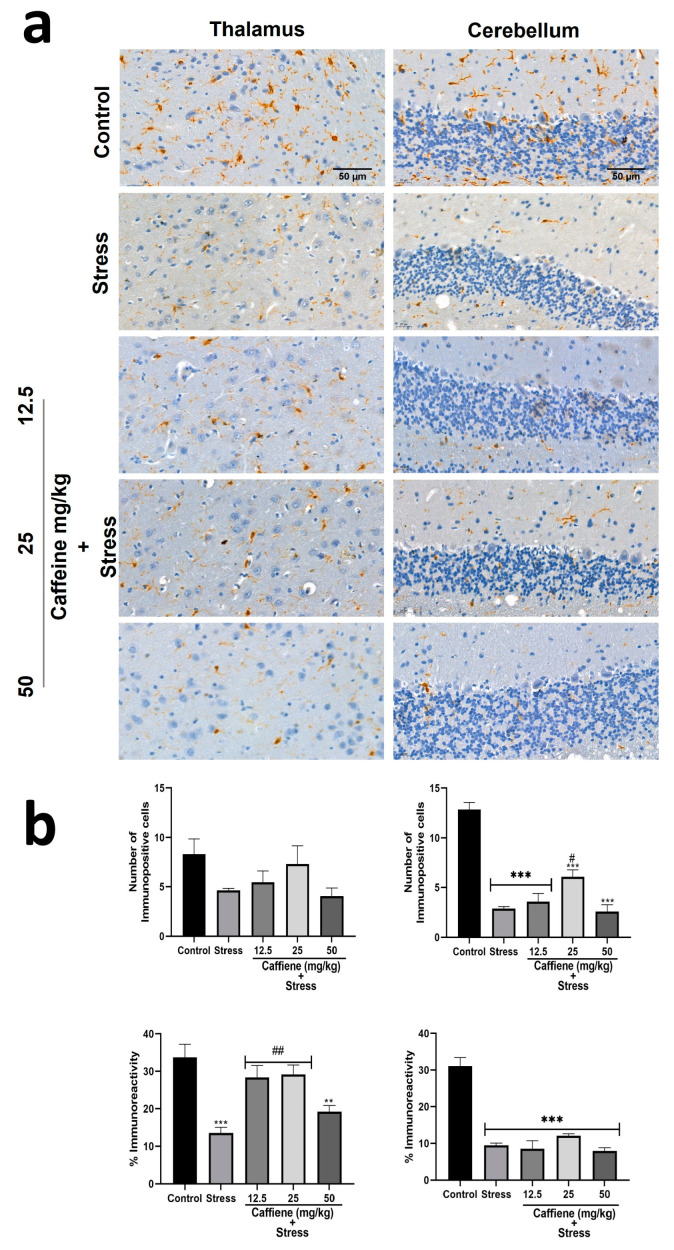
Comparison of the influence of caffeine administration on Iba-1 immunoreaction in the thalamus and cerebellum of UCMS-exposed rats. (**a**) Representative photomicrographs (magnification = ×400). (**b**) Bar graphs showing immunoreactivity (down) and the number of Iba-1-positive cells (up). Each bar denotes group mean ± SEM., ** *p* < 0.01, *** *p* < 0.001 vs. control; # *p* < 0.05, ## *p* < 0.01, vs. stress. One-way ANOVA with Tukey’s post hoc test.

In the thalamus, one-way ANOVA showed significant differences between the groups for percentage immunoreactivity (F (4, 15) = 10.15; *p* = 0.0003) (Figure 14). Post hoc tests revealed a decrease in Iba-1 percentage immunoreactivity in the stress group when compared to the control. This was reversed by caffeine (12.5 and 50 mg/kg) treatment.

In the cerebellum, one-way ANOVA showed a significant difference between the treatment groups: the number of immunopositive cells (F (4, 15) = 42.28; *p* < 0.0001) and percentage immunoreactivity (F (4, 15) = 40.66; *p* < 0.0001) (Figure 14). Post hoc analysis revealed a decreased number of Iba-1 positive cells in the UCMS-exposed rats in comparison to the non-stress control. This effect was significantly reversed by caffeine (25 mg/kg) administration. The UCMS-exposed rats also showed decreased Iba-1 immunoreactivity in comparison to the control. However, caffeine administration produced no effect to counteract this change.

## 4. Discussion

The deleterious impacts of chronic stress on various organ systems have been demonstrated by previous studies [38,39,40,41,42]. Here, the effects of caffeine on these chronic stress-induced alterations were investigated. Stress-responsive parameters were assessed in UCMS-exposed rats and they were compared against caffeine-treated rats exposed to UCMS. The results showed that caffeine administration mostly protected against the effects of chronic stress.

The use of fur appearance to evaluate depressive-like behavior is based on the auto-grooming behavior of rodents, which is carried out to maintain physical hygiene [13,43]. In a depressive-like state, this behavior tends to decrease and may be reflected in the fur’s appearance as a deteriorated coat state. We found that UCMS worsened the coat state, as indicated by the increased fur appearance score compared to the control. This was expected as auto-grooming behavior is responsive to aversive situations like stress [13]. The deterioration of the coat state can be due to decreased grooming behavior, which may be a consequence of disturbance of self-directed behavior [8,38]. The improvement in the coat state of the caffeine-treated groups might be related to caffeine’s effects on mood as it is known to cause arousal in low mood states and provide temporary relief against minor fatigue.

The sucrose preference test assesses for anhedonia, a symptom of depression. The decreased sucrose preference observed in the stress group, which is indicative of anhedonia, agrees with previous studies [2,5,43,44] as UCMS is known to induce a depressive-like state. Caffeine administration reversed this effect as the caffeine-treated groups had increased sucrose preference compared to the stress group. This suggests the anti-depressant activity of caffeine to relieve stress-induced depression.

The use of a forced swim test to assess depressive-like behavior is based on the idea that immobility reflects behavioral despair, and decreased active behaviors such as struggling and swimming indicate depressive-like behavior [45,46]. The increase in immobility time observed in the stress group was consistent with other studies [47,48] as UCMS induces alterations that are linked with endogenomorphic depression such as elevated corticosteroid levels, inability to respond to a reward stimulus, and a lack of reactivity to acute stress [44]. The study also showed that stress control had decreased mobility time and active swimming time. The decreased immobility time observed in the caffeine-treated groups correlates with a previous study carried out by Pechlivanova et al. [48], but contrary to that study, the effect observed was not dose dependent as the 25 mg/kg caffeine produced the largest decrease in immobility time. The finding that caffeine increases active swimming time and mobility suggests that it might possess antidepressant effects.

In the EPM test for anxiety, the finding that UCMS caused increased latency, increased duration in the closed arm, and decreased duration in the open arm, all of which reflect anxiety-like behavior, was expected as studies show that prolonged exposure to stress can lead to anxiety [49]. The results also show that caffeine intake reversed these effects, which could be interpreted as caffeine being anxiolytic. However, this contradicts common knowledge that caffeine is known to be anxiogenic. A closer inspection of the results shows that slightly lower doses of caffeine (12.5 and 25 mg/kg) produced a greater effect at relieving anxiety-like behaviors, suggesting that caffeine might be anxiolytic when consumed in small doses.

In the light and dark test, anxiety-like behaviors were reflected as increased latency time, increased duration in the dark compartment, and decreased duration in the light compartment. Similar to the previous test, UCMS caused increased anxiety-like behaviors, which were slightly reversed by caffeine intake. The results show that smaller doses of caffeine tend to produce a greater effect in reducing these behaviors. This suggests that caffeine could be anxiolytic when consumed in small doses.

UCMS decreased memory performance in the Y-maze test and object recognition test, as shown by decreased percentage alternation in the Y-maze test, decreased time spent with the novel object, and lower preference score in the ORT. These findings suggest impairment of spatial memory and recognition memory, respectively, which could be explained by alterations in the structure and function of the hippocampus following exposure to chronic mild stress [50]. It has been reported that decreased hippocampal volume and dysregulation of corticosteroid levels and brain-derived neurotropic factors could lead to impaired spatial cognition [51]. The finding that caffeine anhydrous administration reversed memory deficits induced by chronic mild stress is consistent with other studies [52,53,54]. This effect can be linked to caffeine’s antagonistic action on A2A receptors [52]. Adenosine A2A receptors play a key role in preventing memory deterioration. This is likely because these receptors are located in synapses in limbic areas, and they can control glutamatergic transmission, NMDA receptor-dependent plasticity, apoptosis, brain metabolism, and neuroinflammation [54].

Our results showed that exposure to chronic stress caused decreased locomotion performance, as evidenced by decreased grades, hanging time in the wire grip test, increased number of foot slips, latency, and time taken to enter the box in the beam walking test. This finding is in agreement with a similar study by George et al. [55]. The alterations were reversed by caffeine administration, particularly the 25 mg/kg dose. This suggests the ability of caffeine anhydrous to ameliorate stress-induced locomotion dysfunctions.

Stress response tends to be reflected in serum corticosterone and blood glucose levels [56]. Exposure to stress causes stimulation of the HPA axis, which causes the release of adrenaline and glucocorticoids from the adrenal gland. The released chemical substances provide the needed energy for the organism to cope with aversive stressful situations [57]. From this study, corticosterone and glucose levels were elevated following exposure to UCMS, as is consistent with previous studies [57,58,59]. This is likely due to increased adrenal cortex secretion of glucocorticoids in response to increased stress levels [60]. Exposure to stressful stimuli stimulates the HPA axis, increasing the release of glucocorticoids, which causes several changes required for coping [61]. Exposure to glucocorticoid in excessive amounts has been shown to cause hyperglycemia [62]. This could explain the increased glucose levels observed in the stressed rats. The finding that caffeine consumption reduced corticosterone and glucose levels suggests that caffeine improved the ability to deal with stress.

Cholinesterases are known for their degradative activity of ACh, a neurotransmitter that plays a key role in memory function [63]. This enzymatic activity has been associated with cognitive dysfunction and cholinergic neuronal damage [64]. In this study, exposure to UCMS elevated levels of cholinesterases in comparison with the non-stress control. Heightened cholinesterase activity promotes ACh depletion, impairing memory function. This might explain the poor performance of the stress-exposed rats in the memory tests (Y-maze test and ORT). The finding that caffeine administration reduced cholinesterase levels is indicative of its positive effects on memory functions.

The current study shows that exposure to the UCMS decreased monoamine levels, as similarly reported by other studies [15,39,65,66,67]. Monoamine depletion has been reported to induce cognitive dysfunction and behavioral depression [68]. Thus, it could be contributing to the depressive-like behaviors observed in the stress-exposed rats. Caffeine administration reversed UCMS depletion of monoamine brain levels, which is in agreement with previous studies [65,66]. This suggests that it might possess anti-depressant properties.

Assessment of kidney function was performed by measuring the level of creatinine, uric acid, and urea in the blood. Elevated levels of these waste products observed in the UCMS-exposed rats are indicative of impaired kidney function. In a similar study carried out by Benchimol de Souza et al. [69], it was found that immobilization stress leads to a lower kidney weight, volume, and glomerular volume density, as well as a lower number of glomeruli per kidney. Thus, these morphological changes in the stressed rats might be responsible for the impaired kidney functions in the rats exposed to UCMS. Caffeine appeared to restore normal kidney functions as the treated rats had lower creatinine, uric acid, and urea levels.

Liver function tests serve as a screening modality for liver dysfunction [70]. They include several biochemical tests for the detection and management of liver diseases. In this study, the levels of liver enzymes including alanine transaminase (ALT), aspartate aminotransferase (AST), and alkaline phosphatase (ALP) were evaluated. Our results show that UCMS significantly increased the levels of all the liver enzymes measured. This finding is consistent with [71]. The elevated level of liver enzymes suggests injury to the hepatocytes, which may be caused by apoptosis of the hepatic cells following exposure to chronic stress. During chronic stress, there is decreased hepatic blood flow, which leads to mitochondria hypoxia. This in turn causes an increase in ROS levels resulting in oxidative stress [72]. The occurrence of oxidative stress, which causes excessive production of inflammatory cells, triggers inflammation and hepatic necrosis [73]. Caffeine administration, especially the 25 mg/kg dose protected against these effects and restored the liver enzyme concentration to normal.

Our findings confirm earlier findings stating the ability of chronic stress to induce oxidative stress [74,75]. Exposure to UCMS reduced the levels of antioxidants including catalase, TAC, SOD, GPx, GST, Na^+^ K^+^-ATPase, and Ca^2+^-ATPase, thus predisposing the rats to oxidative stress. However, it did not influence H^+^K^+^-ATPase, nitrate, and nitrite levels. The elevated MDA levels in the stress group are indicative of lipid peroxidation, which causes damage to the tissues. Treatment with lower doses of caffeine reversed UCMS-induced changes in TAC, GPx, and GST levels, suggesting the potential antioxidant properties of caffeine.

GFAP is an intermediate filament protein found in astrocyte cytoskeleton. It is often used as a marker to assess the astroglial population in brain tissue. In this study, we found that chronic stress reduced the astroglial population in the cerebellum, prefrontal cortex, and CA1 zone of the hippocampus. Similar outcomes have been reported in other studies [76,77,78]. The loss of glial cells in the prefrontal cortex may contribute to the onset of depression as postmortem studies show reduced density and population of glial cells in the front-limbic brain region of depressed patients [79]. Along with the major brain areas recognized to be associated with stress, research also indicates that the thalamus reflects brain activity related to stress management [80], and that the cerebellum also exhibits the mechanisms needed to handle stress-related neurochemical mediators [81]. Treatment with caffeine mildly increased the number of GFAP-positive astrocytes but did not restore them to their original levels. It also reversed stress-induced changes in GFAP expression in the cerebellum. Microglial cells were assessed using Iba-1. The extent of its expression serves as the basis for estimating the microglial population. Chronic stress reduced the microglial population in the cerebellum, prefrontal cortex, and hippocampus (CA1 and DG), as similarly reported by Kreisel et al. [82], although some other studies produced contrasting outcomes [83,84]. Caffeine (12.5 and 25 mg/kg) administration reversed this effect only in the cerebellum and CA1 zone of the hippocampus.

## 5. Conclusions

This study shows that long-term exposure to stress can produce a wide range of effects, altering behavior, cognition, organ functions, neurotransmitter activities, and neuroinflammatory responses. Treatment with caffeine anhydrous reversed most of these changes, suggesting its amelioratory potential against chronic stress-induced alterations. These activities may include mechanisms related to antioxidation, inhibition of corticosterone and anticholinesterase enzymes, neuroprotection, and glial protection. Although its effects were not dose dependent, 25 mg/kg doses consistently produced the best outcome in reducing the aversive effects of chronic stress.

## Figures and Tables

**Table 1:** UCMS schedule.

Week	Session	Days						
		1	2	3	4	5	6	7
1	Morning	Water deprivation (6 h)	No beddings (2 h)	Hypoxia (15 m)	Forced swim (6 m)	Foreign object (24 h)	Shaking (1 h)	Predator sound (2 h)
	Afternoon	Tilted cage (1 h)	Food deprivation (12 h)	Wet cage (2 h)	White noise (2 h)		Damp bedding with no food or water (12 h)	Hot air (5 m)
2	Morning	Tilted cage (1 h)	Tail suspension (6 m)	Hot air (5 m)	White noise (2 h)	Foreign object (24 h)	Predator sound (2 h)	Shaking (1 h)
	Afternoon	Food deprivation (12 h)	Forced swim (6 m)	Food deprivation (12 h)	Wet cage (2 h)		Damp bedding with no food or water (12 h)	No beddings (2 h)

‘h’ means hours and ‘m’ means minutes.

## Data Availability

Data supporting the study can be made available by the authors upon reasonable request. The data are not publicly available due to privacy concerns.

## References

[1] Kyrou I., Tsigos C. (2009). Stress hormones: Physiological stress and regulation of metabolism. Curr. Opin. Pharmacol..

[2] Ahmed A.O., Mantini A.M., Fridberg D.J., Buckley P.F. (2015). Brain-derived neurotrophic factor (BDNF) and neurocognitive deficits in people with schizophrenia: A meta-analysis. Psychiatry Res..

[3] Mariotti A. (2015). The effects of chronic stress on health: New insights into the molecular mechanisms of brain–body communication. Future Sci. OA.

[4] Smyth J., Zawadzki M., Gerin W. (2013). Stress and disease: A structural and functional analysis. Soc. Personal. Psychol. Compass.

[5] Senanayake G.B., Arambepola C. (2019). Understanding chronic stress: A narrative review of literature. J. Coll. Community Physicians Sri Lanka.

[6] Chu B., Marwaha K., Sanvictores T., Ayers D. (2021). Physiology, stress reaction. StatPearls.

[7] Pagliaccio D., Barch D.M. (2019). Stress and the Brain. The Oxford Handbook of Stress and Mental Health.

[8] Wang X., Michaelis E.K. (2010). Selective neuronal vulnerability to oxidative stress in the brain. Front. Aging Neurosci..

[9] Çalışkan G., Müller A., Albrecht A. (2020). Long-term impact of early-life stress on hippocampal plasticity: Spotlight on astrocytes. Int. J. Mol. Sci..

[10] Sugama S., Kakinuma Y. (2021). Noradrenaline as a key neurotransmitter in modulating microglial activation in stress response. Neurochem. Int..

[11] Matović V., Buha A., Ðukić-Ćosić D., Bulat Z. (2015). Insight into the oxidative stress induced by lead and/or cadmium in blood, liver and kidneys. Food Chem. Toxicol..

[12] Juliano L., Ferré S., Griffiths R. (2009). Caffeine: Pharmacology and Clinical Effects. Principles of Addiction Medicine.

[13] Aluko O.M., Umukoro S. (2020). Role of purinergic signaling pathways in the adaptogenic-like activity of methyl jasmonate in rats exposed to unpredictable chronic mild stress. Drug Metab. Pers. Ther..

[14] Liu M.-Y., Yin C.-Y., Zhu L.-J., Zhu X.-H., Xu C., Luo C.-X., Chen H., Zhu D.-Y., Zhou Q.-G. (2018). Sucrose preference test for measurement of stress-induced anhedonia in mice. Nat. Protoc..

[15] Aluko O.M., Umukoro S. (2020). Methyl jasmonate reverses chronic stress-induced memory dysfunctions through modulation of monoaminergic neurotransmission, antioxidant defense system, and Nrf2 expressions. Naunyn-Schmiedeberg’s Arch. Pharmacol..

[16] Arrant A.E., Schramm-Sapyta N.L., Kuhn C.M. (2013). Use of the light/dark test for anxiety in adult and adolescent male rats. Behav. Brain Res..

[17] Eduviere A.T., Umukoro S., Aderibigbe A.O., Ajayi A.M., Adewole F.A. (2015). Methyl jasmonate enhances memory performance through inhibition of oxidative stress and acetylcholinesterase activity in mice. Life Sci..

[18] Van Putten M., Aartsma-Rus A., Louvain-la-Neuve L. (2011). The Use of Hanging Wire Tests to Monitor Muscle Strength and Condition over Time.

[19] Drucker-Colın R. (2010). Beam walking test. Encycl. Mov. Disord..

[20] Ellman G.L., Courtney K.D., Andres V., Featherstone R.M. (1961). A new and rapid colorimetric determination of acetylcholinesterase activity. Biochem. Pharmacol..

[21] Heinegård D., Tiderström G. (1973). Determination of serum creatinine by a direct colorimetric method. Clin. Chim. Acta.

[22] Kono H., Chen C.-J., Ontiveros F., Rock K.L. (2010). Uric acid promotes an acute inflammatory response to sterile cell death in mice. J. Clin. Investig..

[23] Reitman S., Frankel S. (1957). A colorimetric method for the determination of serum glutamic oxalacetic and glutamic pyruvic transaminases. Am. J. Clin. Pathol..

[24] Wright P., Leathwood P., Plummer D. (1972). Enzymes in rat urine: Alkaline phosphatase. Enzymologia.

[25] Bradford M.M. (1976). A rapid and sensitive method for the quantitation of microgram quantities of protein utilizing the principle of protein-dye binding. Anal. Biochem..

[26] Sinha A.K. (1972). Colorimetric assay of catalase. Anal. Biochem..

[27] Misra H.P., Fridovich I. (1972). The role of superoxide anion in the autoxidation of epinephrine and a simple assay for superoxide dismutase. J. Biol. Chem..

[28] Moron M.S., Depierre J.W., Mannervik B. (1979). Levels of glutathione, glutathione reductase and glutathione S-transferase activities in rat lung and liver. Biochim. Biophys. Acta (BBA)-Gen. Subj..

[29] Maral J., Puget K., Michelson A. (1977). Comparative study of superoxide dismutase, catalase and glutathione peroxidase levels in erythrocytes of different animals. Biochem. Biophys. Res. Commun..

[30] Totan Y., Çekiç O., Borazan M., Uz E., Sögüt S., Akyol Ö. (2001). Plasma malondialdehyde and nitric oxide levels in age related macular degeneration. Br. J. Ophthalmol..

[31] Bories P.N., Bories C. (1995). Nitrate determination in biological fluids by an enzymatic one-step assay with nitrate reductase. Clin. Chem..

[32] Ohkawa H., Ohishi N., Yagi K. (1979). Assay for lipid peroxides in animal tissues by thiobarbituric acid reaction. Anal. Biochem..

[33] Wallmark B., Larsson H., Humble L. (1985). The relationship between gastric acid secretion and gastric H+, K+-ATPase activity. J. Biol. Chem..

[34] Bonting S. (1970). Sodium-potassium activated adenosine triphosphatase and cation transport. Membr. Ion Transp..

[35] Hjertén S., Pan H. (1983). Purification and characterization of two forms of a low-affinity Ca^2+^-ATPase from erythrocyte membranes. Biochim. Et Biophys. Acta (BBA)-Biomembr..

[36] Ijomone O.M., Olatunji S.Y., Owolabi J.O., Naicker T., Aschner M. (2018). Nickel-induced neurodegeneration in the hippocampus, striatum and cortex; an ultrastructural insight, and the role of caspase-3 and α-synuclein. J. Trace Elem. Med. Biol..

[37] Paxinos G., Watson C. (2007). The Rat Brain in Stereotaxic Coordinates.

[38] Ushijima K., Morikawa T., To H., Higuchi S., Ohdo S. (2006). Chronobiological disturbances with hyperthermia and hypercortisolism induced by chronic mild stress in rats. Behav. Brain Res..

[39] Pechlivanova D.M., Tchekalarova J.D., Alova L.H., Petkov V.V., Nikolov R.P., Yakimova K.S. (2012). Effect of long-term caffeine administration on depressive-like behavior in rats exposed to chronic unpredictable stress. Behav. Pharmacol..

[40] Paolo S.D., Peana A.T., Carboni V., Serra G. (2000). Exploratory behaviour and grooming after repeated restraint and chronic mild stress: Effect of desipramine. Eur. J. Pharmacol..

[41] Marin M.-F., Lord C., Andrews J., Juster R.-P., Sindi S., Arsenault-Lapierre G., Fiocco A.J., Lupien S.J. (2011). Chronic stress, cognitive functioning and mental health. Neurobiol. Learn. Mem..

[42] McEwen B.S. (2008). Central effects of stress hormones in health and disease: Understanding the protective and damaging effects of stress and stress mediators. Eur. J. Pharmacol..

[43] Nollet M., Guisquet A.M.L., Belzung C. (2013). Models of depression: Unpredictable chronic mild stress in mice. Curr. Protoc. Pharmacol..

[44] Willner P., Mitchell P. (2002). The validity of animal models of predisposition to depression. Behav. Pharmacol..

[45] Yankelevitch-Yahav R., Franko M., Huly A., Doron R. (2015). The forced swim test as a model of depressive-like behavior. J. Vis. Exp..

[46] Lam V.Y., Raineki C., Takeuchi L.E., Ellis L., Woodward T.S., Weinberg J. (2018). Chronic stress alters behavior in the forced swim test and underlying neural activity in animals exposed to alcohol prenatally: Sex-and time-dependent effects. Front. Behav. Neurosci..

[47] Meyer L., Caston J. (2004). Stress alters caffeine action on investigatory behaviour and behavioural inhibition in the mouse. Behav. Brain Res..

[48] Pechlivanova D., Tchekalarova J., Nikolov R., Yakimova K. (2010). Dose-dependent effects of caffeine on behavior and thermoregulation in a chronic unpredictable stress model of depression in rats. Behav. Brain Res..

[49] Ardayfio P., Kim K.-S. (2006). Anxiogenic-like effect of chronic corticosterone in the light-dark emergence task in mice. Behav. Neurosci..

[50] Bakhtiari-Dovvombaygi H., Izadi S., Zare M., Asgari Hassanlouei E., Dinpanah H., Ahmadi-Soleimani S.M., Beheshti F. (2021). Vitamin D3 administration prevents memory deficit and alteration of biochemical parameters induced by unpredictable chronic mild stress in rats. Sci. Rep..

[51] Nair A., Vadodaria K.C., Banerjee S.B., Benekareddy M., Dias B.G., Duman R.S., Vaidya V.A. (2007). Stressor-specific regulation of distinct brain-derived neurotrophic factor transcripts and cyclic AMP response element-binding protein expression in the postnatal and adult rat hippocampus. Neuropsychopharmacology.

[52] Kaster M.P., Machado N.J., Silva H.B., Nunes A., Ardais A.P., Santana M., Baqi Y., Müller C.E., Rodrigues A.L.S., Porciúncula L.O. (2015). Caffeine acts through neuronal adenosine A2A receptors to prevent mood and memory dysfunction triggered by chronic stress. Proc. Natl. Acad. Sci. USA.

[53] Alzoubi K., Abdul-Razzak K., Khabour O., Al-Tuweiq G., Alzubi M., Alkadhi K.A. (2013). Caffeine prevents cognitive impairment induced by chronic psychosocial stress and/or high fat–high carbohydrate diet. Behav. Brain Res..

[54] Cunha R.A., Agostinho P.M. (2010). Chronic caffeine consumption prevents memory disturbance in different animal models of memory decline. J. Alzheimer’s Dis..

[55] George S.D., Archana R., Parasuraman S. (2022). Caloric vestibular stimulation induced enhancement of behavior and neurotrophic factors in chronic mild stress induced rats. Front. Pharmacol..

[56] Anisman H., Zacharko R.M. (1990). Multiple neurochemical and behavioral consequences of stressors: Implications for depression. Pharmacol. Ther..

[57] Umukoro S., Aluko O.M., Eduviere A.T., Owoeye O. (2016). Evaluation of adaptogenic-like property of methyl jasmonate in mice exposed to unpredictable chronic mild stress. Brain Res. Bull..

[58] Pitman D.L., Ottenweller J.E., Natelson B.H. (1988). Plasma corticosterone levels during repeated presentation of two intensities of restraint stress: Chronic stress and habituation. Physiol. Behav..

[59] Lowrance S.A., Ionadi A., McKay E., Douglas X., Johnson J.D. (2016). Sympathetic nervous system contributes to enhanced corticosterone levels following chronic stress. Psychoneuroendocrinology.

[60] Berger I., Werdermann M., Bornstein S.R., Steenblock C. (2019). The adrenal gland in stress–Adaptation on a cellular level. J. Steroid Biochem. Mol. Biol..

[61] Marin M.T., Cruz F.C., Planeta C.S. (2007). Chronic restraint or variable stresses differently affect the behavior, corticosterone secretion and body weight in rats. Physiol. Behav..

[62] Kuo T., McQueen A., Chen T.-C., Wang J.-C. (2015). Regulation of glucose homeostasis by glucocorticoids. Glucocorticoid Signal. Mol. Mice Man.

[63] Haam J., Yakel J.L. (2017). Cholinergic modulation of the hippocampal region and memory function. J. Neurochem..

[64] Loizzo M.R., Tundis R., Menichini F., Menichini F. (2008). Natural products and their derivatives as cholinesterase inhibitors in the treatment of neurodegenerative disorders: An update. Curr. Med. Chem..

[65] Ahmad A., Rasheed N., Banu N., Palit G. (2010). Alterations in monoamine levels and oxidative systems in frontal cortex, striatum, and hippocampus of the rat brain during chronic unpredictable stress. Stress.

[66] Nehlig A., Daval J.-L., Debry G. (1992). Caffeine and the central nervous system: Mechanisms of action, biochemical, metabolic and psychostimulant effects. Brain Res. Rev..

[67] Di Chiara G., Loddo P., Tanda G. (1999). Reciprocal changes in prefrontal and limbic dopamine responsiveness to aversive and rewarding stimuli after chronic mild stress: Implications for the psychobiology of depression. Biol. Psychiatry.

[68] Nutt D.J. (2008). Relationship of neurotransmitters to the symptoms of major depressive disorder. J Clin Psychiatry.

[69] Benchimol de Souza D., Silva D., Marinho Costa Silva C., Barcellos Sampaio F.J., Silva Costa W., Martins Cortez C. (2011). Effects of immobilization stress on kidneys of Wistar male rats: A morphometrical and stereological analysis. Kidney Blood Press. Res..

[70] Thapa B., Walia A. (2007). Liver function tests and their interpretation. Indian J. Pediatr..

[71] Jia H.-m., Li Q., Zhou C., Yu M., Yang Y., Zhang H.-w., Ding G., Shang H., Zou Z.-m. (2016). Chronic unpredictive mild stress leads to altered hepatic metabolic profile and gene expression. Sci. Rep..

[72] Tseilikman V., Kozochkin D., Synitsky A., Sibiriak S., Tseilikman O., Katashinsky E., Nikitina A., Vinogradov D., Simbirtsev A. (2012). Does stress-induced release of interleukin-1 cause liver injury?. Cell. Mol. Neurobiol..

[73] Rizwan S., ReddySekhar P., MalikAsrar B. (2014). Reactive oxygen species in inflammation and tissue injury. Antioxid. Redox Signal..

[74] Samarghandian S., Farkhondeh T., Samini F., Borji A. (2016). Protective effects of carvacrol against oxidative stress induced by chronic stress in rat’s brain, liver, and kidney. Biochem. Res. Int..

[75] Djordjevic J., Djordjevic A., Adzic M., Niciforovic A., Radojcic M.B. (2010). Chronic stress differentially affects antioxidant enzymes and modifies the acute stress response in liver of Wistar rats. Physiol. Res..

[76] Banqueri M., Méndez M., Gómez-Lázaro E., Arias J.L. (2019). Early life stress by repeated maternal separation induces long-term neuroinflammatory response in glial cells of male rats. Stress.

[77] Tynan R.J., Beynon S.B., Hinwood M., Johnson S.J., Nilsson M., Woods J.J., Walker F.R. (2013). Chronic stress-induced disruption of the astrocyte network is driven by structural atrophy and not loss of astrocytes. Acta Neuropathol..

[78] Asari M.A., Nawi F.M., Mohd Amin M.S.I., Yusof N.A.M., Sirajudeen K. (2023). Changes to GFAP Immunoreactive Astrocytes in Medial Prefrontal Cortex Following Exposure to Chronic Stress and Antioxidant Supplementation in Rat Model. Malays. J. Med. Health Sci..

[79] Rajkowska G., Miguel-Hidalgo J. (2007). Gliogenesis and glial pathology in depression. CNS Neurol. Disord. Drug Targets.

[80] McEwen B.S., Gianaros P.J. (2010). Central role of the brain in stress and adaptation: Links to socioeconomic status, health, and disease. Ann. N. Y. Acad. Sci..

[81] Moreno-Rius J. (2019). The cerebellum under stress. Front. Neuroendocrinol..

[82] Kreisel T., Frank M., Licht T., Reshef R., Ben-Menachem-Zidon O., Baratta M., Maier S., Yirmiya R. (2014). Dynamic microglial alterations underlie stress-induced depressive-like behavior and suppressed neurogenesis. Mol. Psychiatry.

[83] Tynan R.J., Naicker S., Hinwood M., Nalivaiko E., Buller K.M., Pow D.V., Day T.A., Walker F.R. (2010). Chronic stress alters the density and morphology of microglia in a subset of stress-responsive brain regions. Brain Behav. Immun..

[84] Calcia M.A., Bonsall D.R., Bloomfield P.S., Selvaraj S., Barichello T., Howes O.D. (2016). Stress and neuroinflammation: A systematic review of the effects of stress on microglia and the implications for mental illness. Psychopharmacology.

